# Cutoff levels for newborn screening of 21-OH deficiency in a Brazilian metropolitan area

**DOI:** 10.1016/j.jped.2025.03.003

**Published:** 2025-04-21

**Authors:** Kallianna Paula Duarte Gameleira, Juliana de Vasconcellos Thomas, Vitor Guilherme Brito de Araújo, Cláudia Vicari Bolognani, Sérgio Eduardo Soares Fernandes, Fábio Ferreira Amorim

**Affiliations:** a Escola Superior de Ciências da Saúde (ESCS), Programa de Pós-Graduação em Ciências da Saúde, Brasília, DF, Brazil; b Secretaria de Saúde do Distrito Federal, Serviço de Referência em Triagem Neonatal do Hospital de Apoio de Brasília, Brasília, DF, Brazil; c Faculdade de Medicina, Escola Superior de Ciências da Saúde (ESCS), Brasília, DF, Brazil; d Universidade de Brasília (UnB), Programa de Pós-Graduação em Ciências da Saúde, Brasília, DF, Brazil

**Keywords:** Congenital adrenal hyperplasia, Neonatal screening, 21-hydroxylase deficiency

## Abstract

**Objective:**

To evaluate the accuracy of neonatal 17-hydroxyprogesterone (N17OHP) levels adjusted for birth weight (BW) and time of the sample collection (TC) and propose optimized cutoff values to improve the effectiveness of newborn screening tests for congenital adrenal hyperplasia (CAH—NBS) programs, utilizing a comprehensive dataset encompassing all newborn screening tests for 21-hydroxylase deficiency (21OHD) conducted over a decade in a Brazilian metropolitan region.

**Methods:**

A cross-sectional study analyzed all CAH—NBS tests from newborns aged 2 to 7 days in the Federal District, Brazil, from January 2012 to September 2022. The accuracy of cutoff values based on the 99.5th percentile (99.5P) for BW and TC was compared to the CAH—NBS program of São Paulo and a threshold of ≥20 mg/dL. New cutoff values were proposed to enhance screening effectiveness.

**Results:**

Among the 340,291 newborns screened, CAH-21OHD was confirmed in 11 cases. The N17OHP cutoff in this sample reduced false positives for neonates ≤ 2500 g but increased them for those > 2500 g The proposed cutoff values based on 99.5P from the sample for neonates ≤ 2500 g, combined with a fixed cutoff ≥ 20 mg/dL for those > 2500 g, showed superior specificity (99.83 %, 95 % CI: 99.81–99.84 %), LR+ (579.16, 95 % CI: 524.23–627.87), PPV (1.84, 95 %CI: 1.70–1.99), and accuracy (99.83 %, 95 %CI: 99.81–99.84 %) than prior criteria.

**Conclusion:**

The proposed 17OHP cutoff strategy effectively reduced false positives, improving specificity, LR+, PPV, and accuracy Thus, it optimized CAH—NBS programs while minimizing unnecessary costs and parental distress.

## Introduction

Congenital adrenal hyperplasia (CAH) is a common autosomal recessive metabolic disorder caused by pathogenic variants in adrenal steroidogenic enzyme genes, leading to enzyme deficiency. Over 95 % of cases result from the impaired conversion of 17-hydroxyprogesterone (17OHP) to 11-deoxycortisol due to 21-hydroxylase (21OH) deficiency, caused by CYP21A2 mutations.^1^, ^2^, ^3^, ^4^

This enzymatic defect reduces cortisol synthesis, triggering excessive corticotropin (ACTH) secretion, adrenal androgen overproduction, and varying aldosterone deficiency. CAH due to 21OH deficiency (CAH-21OHD) presents as a spectrum, ranging from severe, life-threatening salt-wasting crises to mild, asymptomatic forms, depending on enzyme impairment.^1^^,^^2^^,^^4^^,^^5^

Historically, CAH-21OHD is classified into classic and nonclassic forms, though CYP21A2 variants exhibit a phenotypic continuum. The classic form, characterized by cortisol deficiency, manifests neonatally and is further divided into salt-wasting (SW) and simple virilizing (SV) subtypes. Global newborn screening (NBS) programs report a classic CAH-21OHD incidence of 1:10,000–1:20,000 live births, with 75 % SW and 25 % SV cases.^3^

Based on newborn screening for CAH (CAH—NBS) programs from different countries, the overall incidence of classic CAH-21OHD is approximately 1:10,000 to 1:20,000 live births. Among these cases, about 75 % present with the SW form and 25 % with the SV form.^3^

To prevent adrenal crises and ensure appropriate sex assignment in affected females, Pang et al.^6^ introduced CAH—NBS using N17OHP in the 1970s. This test facilitates early diagnosis and steroid therapy initiation, preventing severe complications. Many countries, including Brazil, have incorporated CAH—NBS into neonatal programs, with Brazil officially adopting it in 2012.^2^^,^^6^

As a newborn screening test, CAH—NBS should be highly sensitive to detect nearly all infants with classical CAH. Therefore, the cutoff values for N17OHP as an indicator of the disease should ideally achieve 100 % sensitivity. However, it is crucial to also maximize the accuracy of the test by selecting an N17OHP cutoff value that provides high specificity while preserving 100 % sensitivity, thus minimizing false-positive results (FPR). In this aspect, various technical factors can potentially limit the accuracy of CAH—NBS, and there are no universally accepted standards for N17OHP cutoff values to stratify infants.^7^ Additionally, the FPR of tests incurs significant monetary and emotional costs.^8^

Notably, N17OHP levels can be elevated in healthy neonates during the first two days of life and in premature and/or ill neonates, which poses a diagnostic challenge.^1^ Various laboratories utilize a series of birth weight (BW) and/or gestational age (GA) adjusted cutoff points to address this challenge and enhance the sensitivity and specificity of CAH—NBS.^7^ Other ways to minimize the FPR tests include second-tier screening using liquid chromatography with tandem mass spectrometry (LC-MS/MS) or genetic testing. However, these methodologies are still scarce and expensive, particularly for middle and low-income countries.^1^

While CAH—NBS has been implemented in most Brazilian states, few studies have evaluated the optimal cutoff of N17OHP in Brazilian newborns. Currently, the Brazilian Department of Health recommends that the N17OHP cutoff values be adjusted according to BW using the 99th percentile from a study conducted by Hayashi et al.,^9^ which analyzed data from the CAH—NBS program in the Brazilian state of São Paulo.^10^ However, the CAH—NBS program of São Paulo has currently adopted the 99.5th percentile (99.5P), adjusted for BW and time of sample collection (TC), aiming to reduce excessive false-positive results, based on a subsequent study by Hayashi.^11^

In this context, the primary objective of this study is to evaluate the accuracy of neonatal N17OHP levels adjusted for BW and TC to propose optimized cutoff values to improve the effectiveness of CAH—NBS programs using a large sample from a CAH—NBS program in a Brazilian metropolitan area. Additionally, the authors aimed to compare these findings with the cutoff values established by the CAH—NBS program of the Brazilian State of São Paulo^11^ and a universal threshold of ≥ 20 mg/dL, irrespective of BW, while proposing optimized cutoff values to enhance the effectiveness of CAH—NBS programs.

## Methods

### Study design

This cross-sectional study included all consecutive CAH—NBS tests performed on newborns between two and seven days of age at public hospital maternities and public healthcare service units in the Federal District (FD), Brazil, from January 2012 to September 2022. Data were retrospectively obtained from the FD Newborn Screening Reference Service/FD Health Department database.

### Patients

The study included all newborns who collected the CAH—NBS test between two and seven days of age in public maternities and public healthcare service units in the FD from January 2012 to September 2022. Newborns were excluded if they did not report birth date and time, BW, and TC for the CAH—NBS test or N17OHP value or if the dried blood spot sample was deemed unsatisfactory for analysis.

The FD encompasses a metropolitan area with a population of 2,469,489, including Brasilia, Brazil's capital. Its public healthcare system comprises 16 hospitals (12 with maternity services), one birth house, 13 emergency units, and 175 primary healthcare units.

### Data collection

The N17OHP values from the CAH—NBS test results were extracted from the FD Newborn Screening Reference Service database. The N17OHP results of the second and third blood samples were also collected if these samples were requested. Other collected variables included birth date and time, BW, sex, prematurity, twinning, transfusion history, corticosteroid use, and the date and TC for the CAH—NBS test.

According to the FD Newborn Screening Program (FD-NBS), all newborns born in public maternity hospitals and the FD birth house had to collect samples before the newborn was discharged. Whole blood specimens were collected from newborns on an NBS card (903-grade paper, Whatman, USA), and dried blood spot samples were shipped daily to the FD-NBS Reference Service laboratory for analysis.

According to the manufacturer's protocols, the N17OHP value was measured using the dissociation-enhanced lanthanide fluorescence immunoassay in automated systems (Revvity, Finland).

Newborns were classified into two groups based on TC: (a) < 72 h and (b) ≥ 72 h, and five groups based on BW: (a) ≤ 1500 g; (b) 1501–2000 g; 2001–2500 g; (d) 2501–4000 g and (e) > 4000 g. Subsequently, the 99.5P of the 17OH values for all CAH—NBS samples within each BW group for newborns < 72 and ≥ 72 h were calculated and defined as the cutoff values for indicating CAH-21OHD to be evaluated in the sample of the present study.

These N17OHP cutoff values were compared with two alternative methods: (a) a fixed threshold of 17OHP ≥ 20ng/mL, irrespective of BW, and (b) cutoff values adjusted for BW and TC, as adopted by the CAH—NBS program of the State of São Paulo, Brazil, following the study by Hayashi^11^ that employed the same technology for 17OHP measurement as utilized in the present study.

Suspicion of CAH-21OHD was triggered by N17OHP levels exceeding the 99.5P cutoff adjusted for BW and TC. In values above twice the 99.8th percentile (99.8P) and/or the second positive result, the newborns were recalled to undergo confirmatory tests. They received a comprehensive clinical evaluation by a pediatric endocrinologist at the FD-NBS Reference Service. The confirmatory diagnosis for CAH-21OHD was established by increased serum 17OHP and androstenedione levels.

The CAH-21OHD was classified into SW and SV forms according to the sodium and potassium levels during the follow-up of patients who had already been diagnosed and were undergoing treatment with hydrocortisone.

Finally, it should be noted that all samples for 21-hydroxylase deficiency (21OHD) testing in the studied region's public health services are sent to the FD-NBS Reference Service. The authors have not identified or received any notifications of cases with late confirmation among the neonates included in the present study.

### Statistical analysis

Quantitative variables were summarized as mean ± standard deviation (SD), median and interquartile range 25 % to 75 % (IQ25 %−75 %), as appropriate. Categorical variables were summarized as numbers and percentages. For N17OHP values, the authors calculated the 99.5P and 99.8P

The authors compared the performance of the N17OHP cutoff values based on the present data, cutoff values of study of Hayashi,^11^ and the fixed value of ≥ 20ng/mL using the following metrics with their 95 % confidence intervals (95 %CI): sensitivity, specificity, accuracy, positive likelihood ratio (LR+), negative likelihood ratio (LR-), predictive positive value (PPV), and predictive negative value (PNV). Based on these findings, the authors proposed new optimized cutoff values.

Statistical analyses were conducted using IBM Statistical Package for the Social Sciences version 20.0 for Mac and Jamovi 2.3.24 (https://www.jamovi.org). The level of statistical significance was set at a two-sided P-value ≤ 0.05.

### Ethics statement

 The Institutional Review Board of the Education and Research Foundation of Health Sciences (FEPECS), Brasília, Federal District, Brazil, approved the study (opinion number 40861820.6.0000.5553) with a waiver of informed consent. Conducted in accordance with the Declaration of Helsinki, the study used anonymized medical records, resulting in aggregate data that precluded participant identification; thus, written consent was deemed unnecessary.

## Results

Between January 2012 and September 2022, 448,285 newborns in the FD underwent screening for CAH-21OHD. The authors excluded 15,037 newborns due to unsatisfactory blood samples or inconsistent data, such as missing birth date and time, BW, or blood sample collection date and time. Among the 433,248 newborns with validated data, 340,291 had blood samples collected for CAH—NBS between two and seven days of age and were included in this study.

Among the 340,291 newborns, the mean TC was 2.43 ± 0.90 days. Most samples were collected in the hospital maternity wards (94.5 %). The median N17OHP level in CAH—NBS was 4.40 ng/mL (IQ25–75 %: 3.20–6.00 ng/mL). CAH-21OHD was confirmed in 11 newborns (0.00003233 %), Supplementary Table 1S.

Supplementary Table 2S presents the characteristics of newborns diagnosed with CAH-21OHD. The SW form was predominant (*n* = 10), with only one newborn having the SV form. The lowest N17OHP value recorded was 23.10 ng/mL, observed in the male child with the SV form. The highest N17OHP value recorded was 487.00 ng/mL.

Table 1 shows the 99.5P and 99.8P adjusted for BW and TC of the N17OHP values of the samples in this study and from the study by Hayashi.^11^ In the present study, the 99.5P and 99.8P values were higher for newborns weighing ≤ 2500 g and lower for newborns with BW > 2500 g compared to those reported by Hayashi.^11^

**Table 1:** 99.5th percentile (99.5P) and 99.8th percentile (99.8P) adjusted for birth weight and time of sample collection of the neonatal 17-hydroxyprogesterone (17OH) values of the samples in our study and from the study by Hayashi.^11^

Sample	< 72 h			≥ 72 h		
	n (%)	P99.5	P99.8	n (%)	P99.5	P99.8
Our study ≤ 1500 g 1501 – 2000 g 2001 – 2500 g 2501 – 4000 g > 4000 g Total	1,491 (0.6) 2,759 (1.1) 14,421 (5.8) 216,626 (87.8) 11,324 (4.6) 246,621 (100.0)	127 70 37 14 13 -	135 85 47 17 15 -	1,536 (1.6) 2,565 (2.7) 7,739 (8.3) 77,150 (82.4) 4,680 (5.0) 93,670 (100.0)	260 85 47 14 11 -	387 107 61 20 14 -
Hayashi et al.^11^ ≤ 1500 g 1501 – 2000 g 2001 – 2500 g > 2500 g Total	466 (0.2) 1,763 (0,8) 13,527 (6.3) 198,336 (92.6) 214,092 (100.0)	110 56 32 17 **-**	120 71 39 20 **-**	3,583 (6.2) 6,502 (11.3) 6,813 (11.8) 40,820 (70.7) 57.718 (100.0)	147 69 48 20 **-**	173 90 66 25 **-**

Table 2 compares the true positive, false positive, false negative, and true negative results in the CAH—NBS using the N17OHP cutoff values based on P99.5 adjusted for BW and TC for all groups using this sample, the Hayashi,^11^ and a cutoff of ≥ 20 ng/mL. No false negative results were observed for the three criteria for N17OHP cutoff values, confirming that all effectively detected all newborns diagnosed with CAH-21OHD. The N17OHP cutoff value based on 99.5P adjusted for BW and TC in this sample had fewer false positives for newborns weighing ≤ 2500 g and higher when compared to newborns with BW > 2500 g than the other two criteria.

**Table 2:** True positive, false positive, false negative, and true negative results in the congenital adrenal hyperplasia newborn screening (CAH—NHS) using the neonatal 17-hydroxyprogesterone (N17OHP) cutoff values based on P99.5th percentile (99.5P) adjusted for birth weight (BW) and time of sample collection (TC) in our sample, 99.5P adjusted for BW and TC by Hayashi,^11^ and fixed cutoff ≥ 20 ng/mL.

Variable	P99.5–Our sample n (%)	P99.5–Hayashi^11^ n (%)	≥20 ng/mL n (%)
True positive, n ( %) Age < 72 h ≤ 1500 g (*n* = 1,491) 1501–2000 g (*n* = 2,759) 2001–2500 g (*n* = 14,421) 2501–4000 g (*n* = 216,626) > 4000 g (*n* = 686) Age ≥ 72 h ≤ 1500 g (*n* = 1,179) 1501–2000 g (*n* = 4,930) 2001–2500 g (*n* = 82,195) 2501–4000 g (*n* = 11,324) > 4000 g (*n* = 4,680) Total	0 (0.0000) 0 (0.0000) 0 (0.0000) 8 (0.0037) 0 (0.0000) 0 (0.0000) 0 (0.0000) 0 (0.0000) 3 (0.0373) 0 (0.0000) 11 (0.0032)	0 (0.0000) 0 (0.0000) 0 (0.0000) 8 (0.0037) 0 (0.0000) 0 (0.0000) 0 (0.0000) 0 (0.0000) 3 (0.0373) 0 (0.0000) 11 (0.0032)	0 (0.0000) 0 (0.0000) 0 (0.0000) 8 (0.0037) 0 (0.0000) 0 (0.0000) 0 (0.0000) 0 (0.0000) 3 (0.0373) 0 (0.0000) 11 (0.0032)
False positive, n ( %) Age < 72 h ≤ 1500 g (*n* = 1,491) 1501–2000 g (*n* = 2,759) 2001–2500 g (*n* = 14,421) 2501–4000 g (*n* = 216,626) > 4000 g (*n* = 686) Age ≥72 h ≤ 1500 g (*n* = 1,179) 1501–2000 g (*n* = 4,930) 2001–2500 g (*n* = 82,195) 2501–4000 g (*n* = 11,324) > 4000 g (*n* = 4,680) Total	8 (0.5366) 14 (0.5074) 76 (0.5270) 1,200 (0.5540) 54 (0.4769) 8 (0.5208) 13 (0.5068) 41 (0.5298) 422 (5.2422) 25 (0.5342) 1,861 (0.5469)	13 (0.8719) 39 (1.4136) 122 (0.8460) 414 (0.1911) 9 (0.0795) 25 (1.6276) 30 (1.1696) 37 (0.4781) 164 (0.2126) 2 (0.0427) 855 (0.2513)	766 (51.3749) 523 (18.9561) 445 (3.0858) 256 (0.1182) 6 (0.0530) 788 (51.3021) 481 (18.7224) 360 (4.6518) 164 (0.2126) 2 (0.0427) 3791 (1,1140)
False negative, n ( %) Age < 72 h ≤ 1500 g (*n* = 1,491) 1501–2000 g (*n* = 2,759) 2001–2500 g (*n* = 14,421) 2501–4000 g (*n* = 216,626) > 4000 g (*n* = 686) Age ≥72 h ≤ 1500 g (*n* = 1,179) 1501–2000 g (*n* = 4,930) 2001–2500 g (*n* = 82,195) 2501–4000 g (*n* = 11,324) > 4000 g (*n* = 4,680) Total	0 (0.0000) 0 (0.0000) 0 (0.0000) 0 (0.0000) 0 (0.0000) 0 (0.0000) 0 (0.0000) 0 (0.0000) 0 (0.0000) 0 (0.0000) 0 (0.0000)	0 (0.0000) 0 (0.0000) 0 (0.0000) 0 (0.0000) 0 (0.0000) 0 (0.0000) 0 (0.0000) 0 (0.0000) 0 (0.0000) 0 (0.0000) 0 (0.0000)	0 (0.0000) 0 (0.0000) 0 (0.0000) 0 (0.0000) 0 (0.0000) 0 (0.0000) 0 (0.0000) 0 (0.0000) 0 (0.0000) 0 (0.0000) 0 (0.0000)
True negative, n ( %) Age < 72 h ≤ 1500 g (*n* = 1,491) 1501–2000 g (*n* = 2,759) 2001–2500 g (*n* = 14,421) 2501–4000 g (*n* = 216,626) > 4000 g (*n* = 686) Age ≥ 72 h ≤ 1500 g (*n* = 1,179) 1501–2000 g (*n* = 4,930) 2001–2500 g (*n* = 82,195) 2501–4000 g (*n* = 11,324) > 4000 g (*n* = 4,680) Total	1,483 (99.4634) 2,745 (99.4926) 14,345(99.4730) 215,418 (99.4424) 11,270 (99.5231) 1,528 (99.4792) 2,552 (99.4932) 7,698 (99.4702) 76,725 (94.7205) 4,655 (99.4658) 338,419 (99.4499)	1,478 (99.1281) 2,720 (98.5864) 14,299 (99.1540) 216,204 (99.8052) 11,315 (99.9205) 1,511 (98.3724) 2,535 (98.8302) 7,702 (99.5219) 76,983 (99.7835) 4,678 (99.9573) 339,425 (99.7455)	725 (48.6251) 2,236 (81.0439) 13,976 (96.9142) 216,362 (99.8781) 11,318 (99.9470) 748 (48.6979) 2,084 (81.2476) 7,379 (95.3482) 76,983 (99.7835) 4,678 (99.9573) 336,489 (99.8827)

Table 3 presents the newly proposed N17OHP cutoff values alongside the corresponding true positive, false positive, false negative, and true negative results. These cutoff values were derived from the 99.5P of the present sample for BW and TC in neonates ≤ 2500 g, combined with a fixed cutoff of ≥ 20 mg/dL for those > 2500 g The newly proposed N17OHP cutoff values demonstrated a reduction in false positives compared to prior criteria and did not yield any false negative results.

**Table 3:** Newly proposed N17OHP cutoff values and corresponding true positive, false positive, false negative, and true negative results.

	Newly proposed N17OHP cutoff value	True Positive n (%)	False Positive n (%)	False Negative n (%)	True negative n (%)
Age < 72 h ≤ 1500 g (*n* = 1,491) 1501–2000 g (*n* = 2,759) 2001–2500 g (*n* = 14,421 > 2500 g (*n* = 217,312) Age ≥ 72 h ≤ 1500 g (*n* = 1,179) 1501–2000 g (*n* = 4,930) 2001–2500 g (*n* = 82,195) > 2500 g (*n* = 16,004) Total	127 70 37 20 260 85 47 20 -	0 (0.0000) 0 (0.0000) 0 (0.0000) 8 (0.0035) 0 (0.0000) 0 (0.0000) 0 (0.0000) 3 (0.0037) 11 (0.0032)	8 (0.5366) 14 (0.5074) 76 (0.5270) 262 (0.1149) 8 (0.5208) 13 (0.5068) 41 (0.5298) 166 (0.2029) 588 (0.1728)	0 (0.0000) 0 (0.0000) 0 (0.0000) 0 (0.0000) 0 (0.0000) 0 (0.0000) 0 (0.0000) 0 (0.0000) 0 (0.0000)	1,483 (99.4634) 2,745 (99.4926) 14,345 (99.4730) 227,680 (99.8816) 1,528 (99.4792) 2,552 (99.4932) 7,698 (99.4702) 81,661 (99.7971) 339,692 (99.8240)

Table 4 compares the performance of the newly proposed N17OHP cutoff values with the N17OHP cutoff values based on 99.5P adjusted for BW and TC in the present sample, Hayashi,^11^ and a fixed cutoff ≥ 20 ng/mL for all BW groups. The newly proposed cutoff values exhibited higher specificity (99.83 %, 95 %CI: 99.81–99.84 %), LR+ (579.16, 95 %CI: 524.23–627.87), PPV (1.84, 95 %CI:1.70–1,99) and accuracy (99.83, 95 %CI: 99.81–99.84 %) compared to the prior criteria. The evaluated criteria had no significant differences in sensitivity, LR- and NPV.

**Table 4:** Performance of congenital adrenal hyperplasia newborn screening (CAH—NHS) using the neonatal 17-hydroxyprogesterone (N17OHP) cutoff values based on 99.5th adjusted for birth weight in our sample, 99.5th adjusted for birth weight by Hayashi et al.,^11^ and a fixed cutoff ≥ 20 ng/mL for all birth weight groups.

Variable	P99.5 Our sample	P99.5 Hayashi et al. (11)	> 20 ng/mL	Newly proposed N17OHP cutoff value
Sensitivity,% (95 % CI)	100.00 (74.12–100.00)	100.00 (74.12–100.00)	100.00 (74.12–100.00)	100.00 (74.12–100.00)
Specificity,% (95 % CI)	99.45 (99.43–99.48)	99.75 (99.73–99.77)	98.89 (98.85–98.92)	99.83 (99.81–99.84)
LR+, value (95 % CI)	182.85 (174.75–191.32)	397.99 (372.22–425.54)	89.76 (86.96–92.65)	579.167 (524.233–627.879)
LR-, value (95 % CI)	0.00	0.00	0.00	0.00
PPV,% (95 % CI)	0.59 (0.56–0.61)	1.27 (1,19–1,36)	0.29 (0.28–0.30)	1.84 (1.70–1,99)
NPV,% (95 % CI)	100.00 (100.00–100.00)	100.00 (100.00–100.00)	100.00 (100.00–100.00)	100.00 (100.00–100.00)
Accuracy,% (95 % CI)	99.45 (99.43–99.48)	99.75 (99.73–99.77)	98.89 (98.85–98.92)	99.83 (99.81–99.84)

95 % CI, 95 % confidence interval; LR+, positive likelihood ratio; LR-, negative likelihood ratio; PPV, predictive positive value; NPV, predictive negative value.

## Discussion

Most cases belong to the SW form, with a ratio of approximately 10:1 compared to the SV form, consistent with the literature.^3^

A common challenge in CAH—NBS is the high rate of FPR, mainly observed in preterm, low-birth-weight, or stressed newborns, often exhibiting moderate increases in N17OHP levels.^3^^,^^12^^,^^13^ In this study, from a database of a metropolitan Brazilian NHS program, the authors compared an N17OHP cutoff level adjusted for BW with Hayashi's study^11^ and a 17OHP cutoff value ≥ 20 ng/mL for all BW.

CAH-21OHD is a rare genetic disease characterized by multiple hormonal imbalances that can lead to life-threatening SW and hypoglycemia in the neonatal period, potentially resulting in fatality if not promptly detected and treated. CAH—NBS has been shown to reduce morbidity and mortality effectively and has been implemented in many countries.^1^, ^2^, ^3^, ^4^ Since 2001, the Brazilian Department of Health has been coordinating the Brazilian Neonatal Screening Program (BNSP), which covers all the Brazilian states. The BNSP initially focused on screening for phenylketonuria and congenital hypothyroidism (phase I). Subsequently, screening for sickle cell disease and other hemoglobinopathies (phase II) and cystic fibrosis (phase III), followed by CAH-21OHD and biotinidase deficiency in 2012 (phase IV).^14^^,^^15^ Among the Brazilian federal units, the Federal District (FD) was among the first to adopt CAH—NBS by the Brazilian public health system in 2011.^16^

In 2018, the Endocrine Society developed a Clinical Practice Guideline for CAH-21OHD and recommended the incorporation of CAH—NBS into all NBS programs. In this respect, the N17OHP level is a sensitive indicator for CAH-21OHD screening. Still, it can be challenging due to its dynamic changes based on specific individual factors or conditions, which lead especially to FPR. This Guideline also emphasized using automated time-resolved dissociation-enhanced lanthanide fluoroimmunoassay (Auto-DELFIA) to improve specificity by removing cross-reacting substances and reducing false positive results. This refinement is attributed to the DELFIA method's pre-buffer containing danazol, which, in conjunction with a highly specific anti-17OHP antibody, significantly improves analytical sensitivity and specificity. Consequently, this approach yields more reliable results compared to traditional radioimmunoassays, which were previously regarded as the gold standard. Furthermore, the Guideline recommended employing a second-tier screen by liquid chromatography-tandem mass spectrometry (LC-MS/MS) over other methods to enhance the PPV of CAH—NBS.^7^

Several studies have investigated multiple indicators, including BW, GA, and TC, to enhance the screening accuracy for CAH—NBS. These studies aim to determine which variable would be most suitable for establishing a more precise cutoff value, ultimately optimizing the performance and reducing false positive results of CAH—NBS.^11^^,^^13^^,^^17^^,^^18^ However, these approaches have only modestly improved screening accuracy, with the PPV remaining low.^18^ Other researchers have also proposed alternative N17OHP cutoff values to boost the PPV, which vary across ethnic and national groups.^19^^,^^20^

In specific NHS programs, two routine samples in dried blood spots are collected to reduce false negative results, as approximately 15 % of SW cases were detected only on the second specimen.^21^^,^^22^ Furthermore, some authors advocate implementing LC-MS/MS as a second-tier method to mitigate false positive results and reduce costs.^23^ However, a study in Minnesota showed some missed cases after second-tier analysis.^22^^,^^24^

The proposed adjusting cutoff values perform better than the prior criteria. There was no significant difference in sensitivity among the three criteria, with all capable of detecting all confirmed cases of CAH-21OHD. The newly proposed N17OHP cutoff values exhibited enhanced specificity, LR+, PPV, and accuracy compared to prior criteria. These improvements are primarily attributed to reduced FPR achieved by adopting optimized cutoff levels, thereby enhancing LR+. Deeks and Altman^25^ emphasized that in clinical practice, it is essential to understand the appropriate use of diagnostic tests, especially when dealing with rare diseases. In such cases, they recommend using likelihood ratios (LR) to calculate the probability of abnormality while adjusting for different prior probabilities based on various contexts. Sensitivities and specificities merely describe how abnormality (or normality) predicts specific test results, and the positive predictive value (PPV) provides probabilities of abnormality, which are contingent on the disease prevalence.^26^

This study has some limitations. First, the data were retrospective. Second, the N17OHP levels may have been influenced by factors not assessed in the present study, such as GA, which may be a limitation. In this context, the authors used BW because this data had been presented on the blood collection card, and GA was not. Third, the N17OHP level can vary depending on the method used for its measurement. Although the LC-MS/MS second-tier assay could potentially reduce FPR, this method is not yet available in the public FD Newborn Screening Reference Service. Additionally, implementing a second screening sample may increase costs, time of results, and stress for parents. Fourth, the authors evaluated CAH—NBS among newborns born in Brazilian public maternity hospitals. For example, most middle- and upper-class Brazilian residents have private health insurance and generally do not use public health system services. Therefore, these results should be interpreted cautiously for populations in other contexts. Although no universally accepted standards exist for stratifying infants with CAH—NBS, the Brazilian Department of Health recommended using BW adjustment to improve CAH—NBS. In this regard, the present sample was one of the largest in Brazil, which may better reflect diagnostic testing due to the rarity of the disease. Additional studies aimed at evaluating the optimal N17OHP cutoff point to minimize false positives without compromising test sensitivity should be conducted to enhance the efficiency of the examination, thereby reducing significant monetary and emotional costs.

CAH—NBS enables early recognition and treatment of the CAH-21OHD. The efficiency of this test was demonstrated in the present study by detecting all cases of CAH-21OHD with the first sample, most of which were collected before the newborns were discharged from the maternity ward. However, the authors encountered challenges related to the quantity of FPR, which have been associated with unnecessary costs for additional tests and emotional stress for parents facing the possibility of their child inheriting a hereditary disease. In the present study, which includes one of the largest sample sizes compared to previous studies in Brazil, the authors identified a higher cutoff value for N17OHP in low birth weight newborns, reducing positive results and consequently impacting the test's specificity, LR+, and accuracy. Moreover, 20ng/ml as a cutoff value for children above 2500 g reduced the FPR without impacting the diagnosis. In this regard, it would benefit other CAH—NBS programs to analyze their data to enhance test performance within their respective populations.

## Funding

This research did not receive any specific grant from funding agencies in the public, commercial, or not-for-profit sectors.

## Conflicts of interest

The authors declare no conflicts of interest.

## Appendix Supplementary materials

Image, application 1
