# Supplementary material for: Cutoff levels for newborn screening of 21-OH deficiency in a Brazilian metropolitan area
Source: J Pediatr (Rio J). 2025 Apr 21;101(4):561–8. doi: 10.1016/j.jped.2025.03.003 (PMC12276588; doi:10.1016/j.jped.2025.03.003)
Supplement: Supplementary file 1 [file mmc1.docx]

**JPED-D-24-00477**

**Supplementary Material**

**Supplementary Table S1** Characteristics of the newborns: 17OH results in congenital adrenal hyperplasia newborn screening and confirmed congenital adrenal hyperplasia due to 21-hydroxylase deficiency (CAH-21OHD).

| **Variable** | **Value**  **(n = 340,291)** |
| --- | --- |
| Age, days  Mean (SD)  Median (IQ25-75%) | 2.43 (0.90)  2.00 (2.00-3.00) |
| Age group, n (%)  < 72 hours  ≥ 72 hours | 246,621 (72,5)  93,670 (27,5) |
| Sex, n (%)  Male  Female  Did not recorded | 171,739 (50.5)  163,254 (48.0)  5,298 (1.5) |
| Collected in hospital, n (%)  Yes  No  Did not recorded | 321,430 (94.5)  18,806 (5.5)  55 (0.02) |
| Corticosteroid, n (%)  Yes  No  Did not recorded | 891 (0.3)  319,604 (93.9)  19,796 (5.8) |
| Twinning, n (%)  Yes  No  Did not recorded | 6,758 (2.0)  305,598 (89.8)  27,935 (8.2) |
| Prematurity, n (%)  Yes  No  Did not recorded | 17,596 (5.2)  304,566 (89.5)  18,129 (5.3) |
| Transfusion, n (%)  Yes  No  Did not recorded | 511 (0.2)  307,959 (90.5)  31,821 (9.4) |
| 17OH value, ng/mL  Mean (SD)  Median (IQ25-75%) | 5.25 (5.72)  4.40 (3.20-6.00) |
| Confirm diagnosis of CAH-21OHD, n (%) | 11 (0.00003233) |

IQ25-75%, interquartile range 25-75%; SD, standard deviation.

**Supplementary Table S2** Characteristics of newborns with a confirmed diagnosis of congenital adrenal hyperplasia due to 21-hydroxylase deficiency (n = 11).

| Patient | Sex | Birthweight  (g) | Sample collection (days after birth) | Sample collected in the maternity | 17OH  (ng/mL) | Clinical form |
| --- | --- | --- | --- | --- | --- | --- |
| 1 | M | 3120 | 3 | Yes | 461.0 | SW |
| 2 | F | 3830 | 3 | Yes | 375.0 | SW |
| 3 | F | 3450 | 2 | Yes | 309.0 | SW |
| 4 | M | 3185 | 2 | Yes | 443.0 | SW |
| 5 | M | 3565 | 2 | Yes | 487.0 | SW |
| 6 | M | 2880 | 4 | No | 218.0 | SW |
| 7 | M | 3905 | 2 | Yes | 413.0 | SW |
| 8 | F | 3190 | 2 | Yes | 331.0 | SW |
| 9 | M | 3325 | 2 | Yes | 23.1 | SV |
| 10 | F | 3415 | 2 | Yes | 213.0 | SW |
| 11 | F | 2770 | 2 | Yes | 176.0 | SW |

17OH, 17-hydroxyprogesterone; F, Female; M, Male; SV, simple virilizing; SW, salt-wasting.
